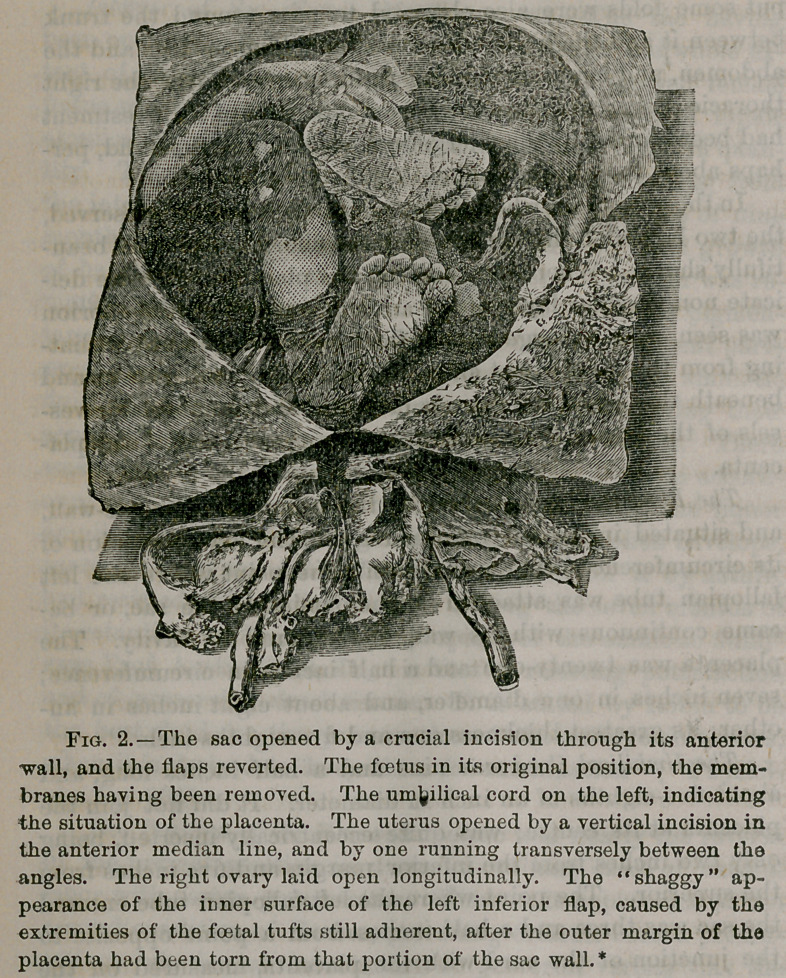# A Case of Full-Term Extra Uterine Gestation of Tubo-Ovarian Form

**Published:** 1876-12

**Authors:** A. Sibley Campbell

**Affiliations:** Augusta, Ga., Demonstrator of Anatomy in the Medical Department of the University of Georgia


ATLANTA
MEDICAL AND SURGICAL JOURNAL.

VOL. XIV.] DECEMBER—1876. [No. 9

### Original Communications.

### A CASE OF FULL-TERM EXTRA UTERINE GESTATION OF THE TUBO-OVARIAN FORM.

WITH SPECIAL EXAMINATION OF THE SAC, UTERUS, AND APPENDAGES.

By A. SIBLEY CAMPBELL, M.D., AUGUSTA, GA.,
Demonstrator of Anatomy in the Medical Department of the University of Georgia.

(With two wood-cuts.)

(*Reprint from American Journal of Obstetrics.*)

The interpretation of phenomena, whether natural or diseased, can be attained only through gradual processes, based upon independent predicates. These must be harvested by many laborers. It therefore becomes the duty of each, into whose path a grain may fall, to preserve it for the reckoning of the future.

For such considerations as these, as well as for its intrinsic interest, I have thought it well to put on record the following remarkable case of extra-uterine gestation; having made the section at the examination after death, removing the pregnant cyst, uterus, and appendages, subjecting them afterwards to a careful study and dissection. The present paper contains the results of my investigations.

"One of the most perplexing questions in the study of a case of extra-uterine pregnancy," says a recent writer, "is that of the class to which it belongs; and this is true after death as well as during life; consequently, the literature of the subject abounds in statements that are utterly unreliable." In my description of this case I have endeavored to give as exact an account of the anatomical condition and relations of the parts interrogated as a special microscopic examination would permit; and if at times I should appear to become prolix, I trust that this will be pardoned on the score of my good intention. I preface my account of the autopsy with a brief relation of the patient's previous history, gathered chiefly from the several physicians under whose more direct observation she chanced to come from time to time. Though not in attendance myself, I had seen her at various times during her stay in the hospital, and the case had become an object of some interest to a number of the profession in this city.

*Previous History.*—K. P., colored, a pluripara, about thirty-three years of age, of medium stature and rather full habit, entered the Freedman's Hospital in this city, November 29th, 1875. She was then supposed to be in premature labor, having experienced during the preceding night, and at the time of her admission, intense pains in the back and abdomen, their character being that of decided uterine efforts; though from her own calculations the full time of her pregnancy would not have expired until about the approaching Christmas. Opiates were administered at appropriate intervals to alleviate her sufferings, and the pains ceased that evening. There was no hemorrhage observed at that time. Subsequent to this attack she was sufficiently improved to leave her bed and to walk about the wards; and did not require any particular treatment, except on two or three occasions, when an anodyne was administered for temporary attacks of abdominal pain of which she complained.

At the time of her admission there was no indication of abnormal gestation, the abdominal tumor corresponding very well in size and general appearance to that of an eight months' pregnancy, in agreement with her supposition of the duration of her gestation. Her general condition had been good, both before and since the beginning of this pregnancy; she had borne three or four children anterior to this time. Her oldest child, a boy fifteen years old, is still living; the youngest died about a year ago, being then about two years old. So that, in her case, there had been no previous condition of a decided and prolonged sterility. During the night of December 21st, she again appeared to have the smptoms of approaching labor, the pains became more decided and intense, and of the nature of expulsory efforts.

In the meantime, Dr. T. R. Wright was in attendance, the conduct of the delivery having been previously assigned him. After this Dr. A. H. Baker also visited the case several times; but the labor did not progress, as there had heretofore been every reason to expect. Prof. Joseph A. Eve and Prof. Henry F. Campbell saw the case in consultation during the continuance of the pains.

The first indication that some abnormal condition existed was founded upon the peculiar action, or non-action of the os and cervix. With the beginning of the pains, or at the time of the first vaginal examination by the gentlemen in attendance, the os was observed to be dilated sufficiently to admit the fore-finger only partially; and this, notwithstanding the decided violence and pertinacity of the pains. During the course of the 22d of December there was some hemorrhage. In the afternoon of the 23d a clot was expelled from the uterus, containing a membranous mass, which could be spread out in a distinct lamina, and when put upon the stretch could be torn like a delicate membrane—as I am informed by Dr. R. O. Gercke, Superintendent of the hospital, to whom, in answer to my inquiries, I am indebted for some of the facts connected with the history of the case. This, I presume, was the decidua—developed within the uterus by the indirect influence of the ectopic pregnancy, as the uterus, in the words of Dr. Parry, "prepares to do its work precisely as if the fertilized germ had entered its cavity"—and its separation the cause of the hemorrhage observed. After the expulsion of this clot a rather fetid discharge, described as containing small pieces of "fleshy matter," was noticed, lasting two or three days, and in quantity like an excessive leucorrhœa. The patient said she had felt the movements of the child up to the morning of the 23d, and I am informed by some of the physicians who saw her at the time that the fœtal heart was heard on the 22d, and observed last about noon of the 23d.

Labor not progressing, and her pain being great, morphine and chloral hydrate were administered, and the pains finally ceased gradually. After the 24th or 25th of December, her general condition became by degrees about the same as before this attempt at parturition. During the pains, which are described as having been in their force and general characteristics identical with those of *bona fide* uterine labor, the vagina was but slightly dilated, and the os and cervix showed but little change in their dilatability from first to last. She is said to have complained during the labor of violent constant pain in the left iliac region. On inquiry, I cannot learn of any indication of menstruation having been observed during her stay in the hospital. About the middle of May, ascites supervened with œdema of the inferior extremities; this becoming worse, respiration was seriously impeded, the œdema probably extending also to the interstitial pulmonary tissue. For this paracentesis abdominis was performed July 20th, by Prof. L. D. Ford, assisted by Dr. G. A. Wilcox, and about two gallons of serum removed from the peritoneal cavity; the subsequent dripping relieved her of a considerable quantity more. After the tapping her respiration was much relieved, and the swelling of the lower limbs diminished. She had now been strictly confined to her bed for three or four weeks; seemed gradually sinking; with the dyspnœa she had great drowsiness—it was difficult to arouse her. The symptoms became decidedly worse during the last two or three days, and she died at 1½ P.M. on the 26th of July, 1876.

*Post-mortem.*—Three hours after death an autopsy was held, there being present Professors Ford, Eve, Campbell, Geddings, Drs. Coleman, Eve, Wilcox, Goodrich, Gercke, Washington, and Mr. Bowers. I had been requested to perform the section and remove the specimen. A longitudinal incision having been made from near the pubes and extending up towards the ensiform cartilage, the sac began at once to protrude beyond the abdominal parietes, rendering it unnecessary to complete the crucial cut as I had expected, and indicating its large size, as well as the small degree to which it was confined to the adjacent viscera and peritoneum. Assisted by Prof. DeSaussure Ford, I separated the sac from the attachments which existed, here and there, between it and the intestines lying behind it. These adhesions were easily torn away from the cyst, by the fingers, with little force. Having removed the few posterior attachments, and having severed also the external borders of the broad ligaments from their continuity with the lateral pelvic peritoneum—taking care not to exclude any of the uterine appendages—the entire mass was held up by two of the gentlemen present, and its full liberation accomplished by a transverse incision through the walls of the vagina, near its junction with the cervix. No appearances of special import were observed in the abdominal cavity. A small amount of dark blood escaping from the uterine and vaginal plexus after their vessels were cut, mixed with some ascitic fluid, was emptied from the subperitoneal pelvic cavity, during the progress of extirpation, as it interfered with a view of the tissues then being severed. The cyst having been removed from the abdomen, and its anterior wall laid open, the fœtus was seen within surrounded with liquor amnii. During my subsequent study of the sac and appendages, I made the following special observations and measurements :

*The Sac* was nearly round in its outline, though not entirely spherical, as its antero-posterior diameter was much shorter than the vertical or transverse, it being moulded upon its contents and by the pressure of its surroundings. This oblate form, however, was determined by the position of the fœtus. Its dimensions—with the fœtus in its original position, and after the liquor amnii had been allowed to escape, which probably caused some shrinkage—were these: transverse circumference, 23¼ inches; vertical circumference, 21¾ inches; oblique circumference, 23 inches; transverse diameter, 9½ inches; antero-posterior diameter, 5 inches; vertical diameter, 8 inches. The external surface was quite smooth, except here and there at points where its suspensory attachments had been torn from it. Near the centre of the posterior aspect, about three inches from its inferior pole, was a patch of white fibrous tissue, its area being two inches vertically by one and a half transversely, very thick, and appearing as compact in structure as the fascicular ligaments. It was no doubt its chief posterior support, probably attaching it to the mesentery. On the anterior superficies six inches from the uterus, a thin, fringe-like membrane, adherent to the sac for two or three inches, grew upward; it was about seven inches in length, highly vascular, and probably nourished the distal zone of the cyst through its capillaries, drawing its supply from an attachment to some of the tissues above.

*The Uterus* did not differ materially in size and conformation from the ordinary unimpregnated multiparous organ. I, however, give the following external measurements: entire length from superior margin of fundus (where attachment of sac began) to anterior lip of cervix, $3\frac{1}{8}$ inches; posterior measurement the same; circumference of fundus, $4\frac{3}{4}$ inches. The os externum and cervical canal were still sufficiently patulous to admit the round handle of an instrument $1\frac{1}{4}$ inch in circumference. Transverse diameter of fundus, $1\frac{1}{2}$ inch; antero-posterior diameter, $1\frac{1}{4}$ inch. Circumference of intra-vaginal portion of cervix, $2\frac{3}{8}$ inches; circumference of supra-vaginal portion, 4 inches. On opening the uterus by a longitudinal incision through its anterior wall and crossing this at right angles by a transverse incision from the uterine extremity of one fallopian tube to that of the other, while the general corporeal cavity and that portion of it leading through the angles to the two fallopian tubes, were found to be contracted as compared with other pariparous* uteri, the os internum, like the os externum, was still somewhat dilated. The mucous membrane was smooth and of a pale red color. Anterior wall in median zone of body, $\frac{7}{16}$ of an inch thick; superior fundal wall, $\frac{5}{16}$ of an inch.

*The Right Fallopian Tube* was $5\frac{1}{4}$ inches in length from its beginning at the right superior angle to the termination of its fimbriated extremity. It was of usual size and normal curve, downwards, backwards, and inwards; its fimbriæ but poorly developed. It was well attached to the right ovary near its termination; not by the fimbriated surface, but by that portion of the tube opposite to the fimbriæ, they being turned outward and away from the ovarium.

*Right Ovary*, $1\frac{1}{2}$ inch long, $\frac{5}{8}$ inch wide, $\frac{7}{16}$ inch thick. Right ovarian ligament, $\frac{5}{8}$ inch long. On section of ovary two small corpora lutea were found, one larger than the other. Just opposite the adhesion of the extremity of the fallopian tube, the larger corpus luteum was $\frac{1}{8}$ inch from the surface. Its section was $\frac{5}{16}$ of an inch long and $\frac{3}{16}$ of an inch wide. The smaller body was $\frac{3}{16}$ inch from the surface of the ovary, and

*I have ventured to introduce a new expression, I believe, as I have just here felt the need of it; not being aware of any single term, now in use, to convey the idea. We already have the adjectives *multiparous, pluriparous, uniparous, primaparous, imparous*, etc., as applied to the present condition, or to the past gestative life of a woman, or of the uterus. By analogy with these, we may have as well, in comparing two or more uteri, to express similarity of past gestative history—in age, number of births, etc.—*pariparous* (*par, paris*, equal, similar; and *pario*, to bear); or if it is preferred, from the Greek, *homotocic* (*omos*, the same; *tokos, partus*, gestation.)

just over it, on the investing membrane, was a deeply-tinged congested spot. The smaller body was about half the size of the larger; both were filled with small white granules, easily removed from their convoluted walls.

*The Left Round Ligament* was about twice as large as the right, the latter being of the usual size. The right round ligament was inserted lower down than the left. While the right tube was inserted into the free right superior angle of the uterus, the junction of the left tube with the uterus was about half an inch higher up, and just under that portion of the sac where it began to rest upon the fundus. The left round ligament had a higher insertion than the junction of the right tube with the uterus. While the left tube began at the uterus a little above the insertion of the round ligament, the right tube began at the same altitude as both its round and ovarian ligaments, the former before and the latter behind it. I mention these slight deviations in symmetry as they have some bearings on the case.

As will be seen from the cuts, the sac rested directly upon the fundus of the uterus. Its principal attachment to that organ was by means of a ligamentous capsular structure extending from the circumference of the fundus to the inferior surface and up the sides of the sac for four or five inches—the inferior zone of the cyst being contained within this cup-shaped ligament—the two together reminding me of the familiar toy of cup and ball, or of a flower in its calyx. This structure was a continuous, thick, ligamentous membrane, extending without any interruption from a little to the left of the median line in front across to the right, thence around the posterior superfices of sac and fundus to a point corresponding to the angle made by the folding down of the left fallopian tube, presently to be described. The calyx then ceased to pass as a continuous lamella from fundus to under surface and side of the sac, as it had up to this point, but its lower portion still continued around from this point towards the front again to the left superior angle of the uterus. After it became incomplete above, it was supplemented by separate bands extending up from the body of the uterus to the sac, and up the sides of the latter, some under and some over the left fallopian tube, the first portion of the tube curving around the left inferior region of the cyst in the interspace where the calyx was some what incomplete. That is, the calyx formed a continuous and complete capsular ligament around the base of the sac through an arc of over 280 degrees. This broad attachment of the sac to the fundus, which I have termed the calyx, was not identical with the proper structure of the sac wall; it could be easily separated in its entire extent by tearing it away with the fingers from the sac. This was not the case in its relations with the fundus; it apparently grew out from its periphery, and had evidently been developed from the uterus, when the sac and contents increasing in size, and their demands for nutrition and support, rolled over from the left iliac region and took its final position upon the fundus. These proliferations were then thrown out, both affording to the pregnant sac a steady support below, and sending blood to the fœtus through its vascular structure, as it was seen to be well provided with capillaries. This ligament could without much difficulty be separated into two laminæ. By its long residence upon the surface of the fundus, that portion of the inferior zone of the sac which rested directly upon it had become slightly adherent to the fundus; but I easily separated the two entirely from each other, showing that there was no communication between the cavity of the uterus and that of the sac, unless we include its possible capillary communication through the ostium uterinum of the left fallopian tube, which from the evidences in the case, was probably closed. The thickest portion of the calyx was in the median line on its posterior aspect, where it had below, near the uterus, a slightly ribbed appearance like a palm-leaf.

*The Left Fallopian Tube.*—Immediately upon the removal of the cyst and contents, with the uterus and appendages from the abdominal cavity, we saw, without further dissection, that an intimate relation had existed between the sac and the left tube. Running up from the left superior angle of the uterus, in a curve whose radius would about equal that of the circle formed by the projection of the sac upon a perpendicular plane, parallel with its posterior aspect, viewing it from before—the tube was situated somewhat to the front and a little beneath the rotundity of the cyst, and attached to it by delicate fibres. The superior extremity of this curve was three inches from the junction of the tube with the fundus. At the first casual examination, the left tube seemed to terminate at the end of this curve, as no more of it could then be seen; and, still more plausibly, because small, reddish fibres, radiating from its extremity and binding it to the sac, appeared as if they might be the fimbriæ, clinging to the sac, and that the cyst might have begun its development at this part of the tube, or at some point intermediate between it and the left angle of the uterus. But I afterwards found an entirely different solution, which involved no element of doubt.

During the subsequent study of the specimen, I made this interesting discovery. Having removed the fascicular attachments running over the left tube, holding it up, in the curve described, to the left inferior region of the sac, and dissecting away from the tube that portion of the left broad ligament also which was necessarily carried up with the tube and lay against the under surface of the sac on the left side, I found that the continuation of the broad ligament outward to the left and somewhat posteriorly, still continued to embrace the under left surface of the sac, and that in the upper margin of this postion of the ligament, *a second portion* of the left fallopian tube existed, heretofore concealed, equal in length with the first portion—that is, three inches—which at its termination entered the cavity of the sac by a canal three lines in diameter.

After leaving the left superior angle of the uterus, the left tube ran around and up the side of the sac, and was attached at the upper extremity of the arc thus formed, as before described. It then became decidedly enlarged beyond its former size—as we should expect, as it continued outward to form the pavilion—and was then folded downwards abruptly upon itself, entirely changing its former direction, the second portion running downwards and slightly backwards to the under surface of the sac, on the left side. The two limbs made an angle with each other of about thirty-five degrees; this angle does not refer to the bend of the tube upon itself, for that was a complete and abrupt folding down and apposition of surfaces, but to the angle formed by the direction of the two after their departure from this point. The second portion of the tube—the *ampoule* of Henle—after extending for three inches downwards and backwards, became continuous with the structure of the sac-wall, just at the point where the tube can no longer be seen in Fig. 1. It was about three-eighths of an inch in diam-

FIG. 1.—Anterior view of the external surface of the sac and its relations. The left fallopian tube curving upwards and outwards, and after bending downwards at an acute angle, abruptly entering the sac towards its under surface. The loose tissues on the left, under that side of the cyst, are the remains of the broad ligament, after dissecting it from the sac, tube, round ligament, and larger vessels. The uterus incised as in Fig. 2.

eter just before it entered the sac; its wall about half a line in thickness. The canal of the first portion, or isthmus of the tube, was still minute, if not closed entirely; its walls seemed atrophied and weak, perhaps from stretching and long pressure of the sac; hence, to explore the second portion, I made an incision into its wall about an inch and a half from the sac, and introduced through this opening the round ivory handle of an aneurism needle. It passed through the tubal canal into the interior of the sac, and still further on for about two inches. Here the instrument passed out of the canal of the tube, which became more delicate within the sac, and was incorporated with its posterior wall. The point where the instrument passed out was under the placenta. The apparent termination of the tube was, therefore, between the attachment of the placenta and the sac-wall, with the structure of which the tube seemed to become blended. The manner in which the tube entered the sac was abrupt, not gradual; there was no appearance of an ovoid enlargement of the tube, but its junction with the sac was like that of a large, round melon with its slender vine.

*The Left Ovary* could not be found. It might be urged by some that it either became bound down to the peritoneum in the left iliac region, after being pressed upon by the weight of the superincumbent pregnant sac, when in the first stages of its development it occupied the left side of the abdomen, and was accidentally left in the cavity during the removal of the cyst and its connections; that it was atrophied by the same pressure, and lost sight of, by reason of its small size and on account of the changes of form and relation in the parts; or became involved in the posterior portion of the calyx; or inclosed in the interior of the sac—provided there had been early rupture of the cyst; or was itself greatly concerned, together with the left tube, in the formation of the sac. These suggestions I at first thought might be topics of some debate. After a thorough investigation, I found that the last was the only tenable explanation—that the gestation belonged to the tubo-ovarian class, which I shall be able to demonstrate beyond all cavil. In my search for some structure that might be recognized as the metamorphosed ovary, or even its ligament, I observed, in a part of the left posterior wing of the calyx, formed by the posterior wing of the left broad ligament, a band of tissue somewhat thicker than that adjacent to it, which also appeared to contain some red fibres. The idea presented itself that this might have been formed from the left ovarian ligament, carried up in the left broad ligament, as it arose to embrace the sac and assist in forming its protection and support; as these ectopic products appropriate so freely and make subservient to their own nutrition, stability, and vitality, whatever natural tissues happen to be within their proximity and influence.

I probed the right fallopian tube its entire length, from the right superior angle of the uterus to the fimbriated extremity, though, as the instrument at hand was too large for the purpose, I had to employ some force. I found it impossible to probe the left tube throughout in the same manner, as its first portion was much more contracted than that of the right tube, and its walls also too weak to sustain the amount of force required in a forcible passage of the probe.

*Interior of Sac, Fœtus, Membranes, etc.*—The sac having been opened by a free vertical incision through the whole extent of its anterior wall, a remarkably well-preserved female fœtus was seen within, bathed in an ample supply of liquor amnii of light brown color—probably a pint and a half in quantity. There was no decidedly unpleasant odor arising from the interior of the sac. The fœtus was evidently one of nine months gestation. The head was provided with a full growth of hair, and the finger nails were perfect. The skin was remarkably well preserved; merely the cuticle with its pigmentary layer had been rubbed off at some of the prominent parts presenting anteriorly, from long maceration in the amniotic fluid, and from handling the sac and contents during their separation and removal from the maternal abdomen. There was not the least tendency to putrefaction, and this in accordance with the preservative qualities attributed to the liquor amnii; the fœtus being shut up within its unbroken envelope, surrounded by the antiseptic fluid provided for it by nature, had resisted putrefactive changes during the seven months of its retention. "An important property of the amniotic fluid," says Prof. Austin Flint, Jr., "is that of resisting putrefaction and of preserving dead tissues. It is stated by Robin to be the best fluid for the preservation of the embryonic tissues, when it is desired to keep them for examination" ("Physiology," p. 904).

*The Position of the Fœtus* in the sac was nearly the reverse of the normal position in utero in ordinary cases of natural labor with vertex presentation. That is, it approximated the second condition of Barnes, preceding difficult breech presentations, whether of the anterior or posterior variety viz.: the abdomino-anterior position, with the legs extending upwards and approaching the face. The head was situated principally in the left side of the fundal zone of the sac; the right shoulder in the right side of the fundal zone, and the other parts disposed as seen in Fig. 2, where the sac is represented, opened by a crucial incision, with the fœtus *in situ.*

*The Membranes*, though generally investing the fœtus, had apparently fallen away from the anterior prominent points after softening and pressure against the abdominal walls of the mother. They still surrounded some portions of the limbs, but some folds were also observed to pass around the trunk between it and the limbs—between the folded-up legs and the abdomen, and again under the right arm and over the right thoracic space; as if this strange arrangement of its investment had been caused by the energetic movements of the child, perhaps about the time of labor.

In those portions where the membranes were best preserved, the two layers of the aminon and the chorion were still beautifully shown. Where they covered the placenta, after the delicate non-vascular layer of the aminon was raised, the chorion was seen, with its large vessels, the size of a crow-quill, radiating from the funis about an inch apart from each other; and beneath this again the densely crowded and innumerable vessels of the fœtal tufts, constituting the great mass of the placenta.

*The Placenta* was attached to the interior of the sac-wall, and situated in its left inferior region; the lowest portion of its circumference being just opposite the point where the left fallopian tube was attached to the outside of the sac, or became continuous with its wall, and entered its cavity. The placenta was twenty-one and a half inches in circumference; seven inches in one diameter, and about eight inches in another; its greatest thickness one and five-eighths inch.

*The Umbilical Cord* was nine and a half inches long, and about five-eighths of an inch in diameter. It did not join the placenta at its centre; was quite eccentrically inserted, being only two inches from the inferior margin and five inches from the superior. The point where the left fallopian tube entered the sac was three and a half inches from a point opposite to the junction of the cord with the placenta, measured on the outer surface. The indications of this eccentric union of the cord with the placenta, the direction in which the symmetry existed, and the proximity of the site of the placenta to the left tube and the usual situation of the left ovary, are obvious.

The placenta was firmly attached to the sac; it required considerable force to tear it away from the wall. The site from which I had removed some portion of it was left rough and shaggy from the broken ends of the tufts of the chorion which still remained adherent to it.

*The Sac* varied in thickness from about the third of a line to one line; average thickness about half a line. It was formed, apparently, of inelastic, fibrous tissue; small capillary vessels were seen running through its structure. Its inner and outer surfaces had nearly the same appearance. In some

*The cuts are from photographs from Usher's Gallery, Augusta, Ga.; to the proprietor and his assistants I am indebted for courtesies received.

portions its wall could be separated into two laminæ. It had the same general appearance to the naked eye as the calyx-like ligament that supported it below, viz.: fibrous tissue, striped with the numerous capillary vessels supplying its structure. These small vessels were much more abundant near and around the uterus, radiating from the direction of the fundus, finally becoming fewer and more widely scattered as we approach the superior pole of the sac.

*Vessels.*—The main trunk of the uterine artery on the left side was still enlarged, and measured three lines in diameter; the corresponding artery on the right side was considerably smaller, measuring about two lines in diameter. The left ovarian artery was also larger than the right. Probing the former with an instrument the size of an ordinary silver probe, from its outer extremity in the left broad ligament, the instrument passed through the main branch to the left superior angle, and then appeared in the open mouth of one of the vessels severed in the vertical section of the uterus, six or eight of which were seen with their gaping canals in the middle muscular layer of the corporeal wall. The branches of the ovarian pampiniform plexus were larger on the left side than on the right; as also were the vaginal and cervical venous plexus on the left. A probe passed through the enlarged main trunk of the left uterine artery stopped at the left superior angle of the fundus, just where the left ovarian artery passed in to supply the inner muscular wall and anastomose with the uterine. No vessels of any important size could be found leading directly to the cyst, as we sometimes see thrown out in the exigency of their requirements, or formed from the exaggeration of small normal branches, to supply abnormal or ectopic growths. Hence the fœtal sac must have been sustained in its early stages of development—when it resided in the left iliac region, and before it had any connection and intimate relations with the uterus—by the pampiniform plexus of the left ovarian artery, supplying the ovary and fallopian tube. After it had taken up its habitat upon the fundus, receiving its strong attachment with the uterus—through the cup-shaped pseudo-membrane, assisted on the left by the broad ligament—and called for an increased supply of blood; this must have been answered through the vascular layer of the calyx, which was well provided with numerous arterioles and small venous branches, somewhat larger, contained in a middle layer, between the two fibrous laminæ constituting the cortical surfaces. So that the thick vascular walls of the uterus—no doubt much enlarged during gestation—must have acted as a great sponge or diverticulum, rapidly receiving the blood through its proper vessels, and thence transmitting it through the vascular layer of the calyx to the pseudo-matrix, which was serving in its stead.

*Weight.*—The sac and contents, after draining off the liquor amnii, with the uterus and appendages, weighed seven pounds and a half. The fœtus alone weighed five pounds and a quarter.

From the foregoing description I think it will now be agreed that of the several classes of extra-uterine fœtation recognized by systematic writers, the present example was plainly of the tubo-ovarian form. Though the extremity of the left tube was in union with the sac-wall, and the canal of its pavilion communicated with its cavity, the cyst was not developed in the continuity of the oviduct—it was not an instance of the purely tubal form. (1.) Because the free portion of the left tube not at all involved in the sac—even after its fimbriated extremity had been contributed, so to speak, to assist in forming the cyst—was still found to be a little longer than the tube on the right side. (2.) The almost spherical shape of the cyst. When formed in the continuity of the tube, the sac is usually ovoid or spindle-shaped; "when formed in the fimbriated extremity, the sac developed partly out of the dilated mouth of the tube, and partly by attachments to neighboring structures, especially the ovary, has usually a spheroidal shape;" and as a corollary to this reason, (3.) The abrupt manner in which the tube joined the sac—not by a gradual dilatation of its calibre, the dimensions changing at once from a tube a little over an inch in circumference to the spheroidal cyst, whose greatest circumference was over twenty-three inches. (4.) The close resemblance, in its general appearance, which the sac bore to an ovarian cyst. (5.) The absence of the left ovary, which indication is greatly strengthened when viewed in connection with the fact, that (6.) The point in the pelvic cavity where the tube joined the sac was the proper situation of the ovary, modified only by the slight deviation caused by the upward and lateral growth of the cyst. (7.) The fact that the left broad ligament embraced and was attached by its posterior wing to the left inferior surface of the sac. (8.) That the gestation went to full term, without any indication at any time—before or after death—that there had been rupture of the cyst. "It may be said generally," remarks Dr. Barnes, "that the sac bursts the earlier, the nearer its seat is to the uterus. The tubo-ovarian sac may not burst until near the ordinary term of uterine gestation, whilst the tubal or interstitial sac usually bursts at dates varying from six weeks to three months." * * * * "The tube," continues the same author, "although consisting of a mucous and a muscular coat, is still adapted to keep pace in growth with the rapid development of the ovum. The adaptation is not simply, as in the case of uterine gestation, obtained by growth of the tube *pari passu* with its contents; the tube is stretched as well, and there comes a time when the stretching exceeds the distensibility of the tube, the sac bursts, and the contents escape into the peritoneal cavity." There having been no previous indication of the hemorrhage and collapse, so marked after ruptures of the cyst, combined with the other evidences, excludes the idea of this being a case of early rupture of the original envelope, resulting in the abdominal form.

The entire disconnection of the uterine extremity of the fallopian tube—so far as any incorporation was concerned—with that portion of the sac against which it lay, together with the independence and easy separation of the inferior zone of the sac and the thick wall of the fundus upon which it rested, precludes also the possibility of an original interstitial gestation.

The peculiar curve and bending of the attached left tube is easily understood, if we imagine the gestation at first going on in the left iliac region; the sac as it increased in size rolling a little forward and then centrally, thus resting upon the tube; contracting adhesions with it, and as its dimensions still further increased, carrying the tube upwards attached to its external surface, in a curve determined by the manner in which it at first happened to rest upon the oviduct. The abrupt bend upon itself, just about the juncture of the isthmus and the ampoule may have existed before conception, as "it is not rare to find the tube doubled up, either before or behind, and bound down by pathological adhesions."*

The comparatively large size of the left round ligament, would seem to indicate the development of its muscular fibres, while acting as a support to maintain the normal position of the organ resisting right lateral version, as it was being borne upon by the enlarging fœtal cyst in the left region of the pelvis, pushing against the left side of the fundus, and thus having a tendency to force it to the right. In the case of Mr. Hutchinson, referred to below, the cyst had succeeded in forcing the uterus over to the right. According to Rainey, the round ligament is rather a muscle than a ligament, consisting principally of striated fibres.

Barnes, Campbell, and Hecker have called attention to the fact that, as in the present example, the left tube has been the one concerned in a considerable majority of cases. The former has offered as an explanation that the left tube is more liable to displacement and compression by the sigmoid flexure lying in close relation to it, and its being often disturbed by feculent accumulations.

There are other topics of interest suggested in the study of this case, which I omit for fear of prolonging the report beyond its proper limits.

As to the question of the propriety of operative interference, I briefly refer to a few eminent authorities.

Mr. Jonathan Hutchinson, senior surgeon to the London Hospital, published in the *Medical Times and Gazette*, August, 1860, a tabulated paper of one hundred and two cases of extra-uterine fœtation. He reports a case (*London Lancet*, July 19, 1873) which in many particulars bears a striking resemblance to the subject of this paper. In his case also the left fallopian tube was the one involved, the broad ligament passed downwards in front of the cyst, and he was unable satisfactorily to identify the remains of the ovary. In his concluding remarks, in view of his experience with his last case, and quoting also from his former treatise the opinions he had formed from a study of the large number of instances he had collected, he says: "Those opinions I may briefly sum up in the following practical rule—that extra-uterine fœtation cysts ought not to

*Barnes on Diseases of Women; chapter, the Fallopian Tubes.

be meddled with in any way, either by puncture or incision, until suppuration has occurred, and an abscess fistula has been formed."

Dr. Campbell, of Edinburgh, "who collected eighty-five cases of extra-uterine gestation, showed that sixty-two recovered, whilst twenty-three died as a direct consequence of the abnormal pregnancy; of the sixty-two in which recovery took place, in twenty-one the fœtus remained quiescent through life for periods varying from four to fifty-six years, and in the rest its removal had been effected by ulceration. He advised that abdominal section should not be performed until after the system had been restored to its unimpregnated condition, and nature had evinced a disposition to remove the extraneous mass." (Barnes: Chapter on Extra-uterine Gestation.)

Dr. Parry's work, just published, on Extra-uterine Pregnancy, based upon the consideration of five hundred cases, is perhaps the most extended treatise and highest authority extant upon the subject of ectopic fœtation.* Not having the work at hand, I quote a summary of his views from the review, by Dr. J. D. Trask, in the *American Journal of Medical Sciences* April, 1876.

"The prospect of saving the life of the child ought not to be taken into consideration, and the primary operation cannot be too emphatically condemned. In proof of this, if we compare the mortality of cases left to nature, with those following primary gastrotomies, that of the former class is 52.65 per cent., while that of the latter is seventy per cent., or 17.35 per cent. greater than if they had been left to nature. The mortality following the secondary operation, that is, months, or even years after the termination of pregnancy, stands 38.88 per cent., or, as compared with those left to nature (52.65 per cent.), 13.77 per cent. in favor of the operation." He agrees with Mr. Hutchinson that the cyst should not be disturbed in any way until suppuration has occurred and a fistula has been formed, as "the operation is thus degraded from the important and dangerous procedure, gastrotomy, to the simple and less dangerous performance of opening a large abscess."

*Extra-uterine Pregnancy: Its Causes, Species, Pathological Anatomy, Clinical History, Diagnosis, Prognosis, and Treatment. By John S. Parry, M.D., Obstetrician to the Philadelphia Hospital, etc., 1876.

The expectant treatment pursued in the present case seems therefore to have been borne out by the above authorities; especially as the post-mortem examination revealed the fact that there had not been any intimate adhesion between the wall of the cyst and the parietal peritoneum. At the same time, had it been possible, during life, to diagnosticate the minute pathological conditions and relations—as to circulation, mode and extent of attachment, and the exact variety of the abnormal pregnancy—which have been brought to light, with all the advantages of a careful study and dissection, after death; there are few, doubtless, who would in this case have condemned the secondary operation.

## Figures and Tables

**Fig. 1. f1:**
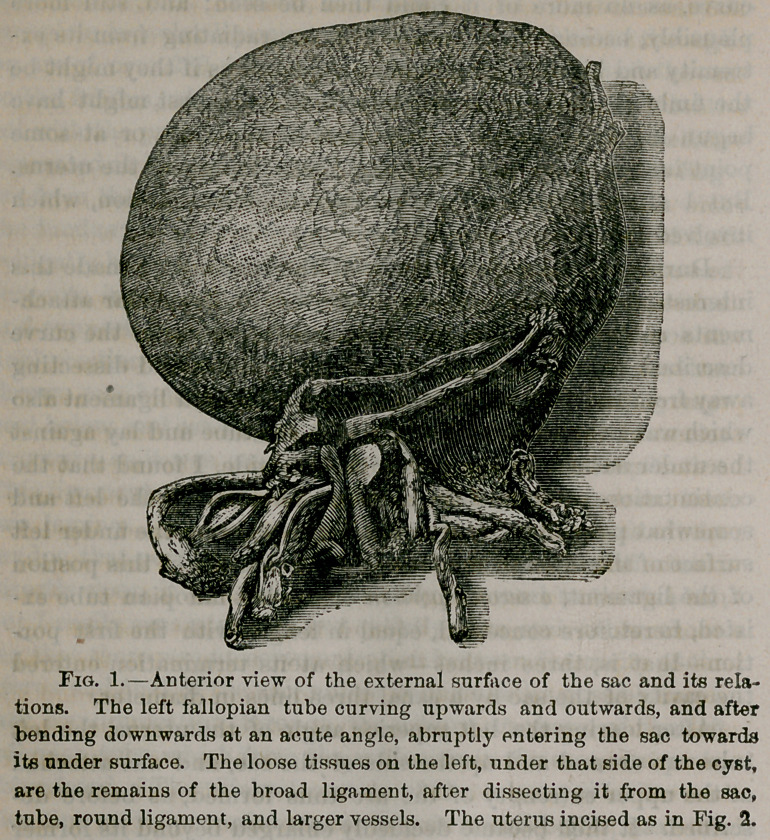


**Fig. 2. f2:**