# Errata

**Published:** 1877-01

**Authors:** 


Errata.—We correct the following more important typographical errors occurring in our publication of Dr. A. Sibley Campbell's article on "A Case of Full-Term Extra-Uterine Gestation of the Tubo-Ovarian Form," in our December number, as one or two of them change entirely the meaning of the author:

Page 514, first line, "microscopic" should be "macroscopic."

Page 518, last line, "*omos*" "*homos.*"

Page 529, twelfth line, "is still adapted" should be "is ill adapted."

Page 530, sixth line, "lateral version" should be "latero-version."

